# Black-White Disparities in Breast Cancer Subtype: The Intersection of Socially Patterned Stress and Genetic Expression

**DOI:** 10.3934/publichealth.2017.5.526

**Published:** 2017-11-24

**Authors:** Erin Linnenbringer, Sarah Gehlert, Arline T. Geronimus

**Affiliations:** 1 Division of Public Health Sciences, Department of Surgery, Washington University School of Medicine, St. Louis, MO 63110-1093, USA; 2 College of Social Work, University of South Carolina, Columbia, SC 29208, USA; 3 Population Studies Center, Institute for Social Research, University of Michigan, Ann Arbor, MI 48106-1248, USA

**Keywords:** breast cancer, health disparities, social environment, social genomics, weathering

## Abstract

Hormone receptor negative (HR-) breast cancer subtypes are etiologically distinct from the more common, less aggressive, and more treatable form of estrogen receptor positive (ER+) breast cancer. Numerous population-based studies have found that, in the United States, Black women are 2 to 3 times more likely to develop HR- breast cancer than White women. Much of the existing research on racial disparities in breast cancer subtype has focused on identifying predisposing genetic factors associated with African ancestry. This approach fails to acknowledge that racial stratification shapes a wide range of environmental and social exposures over the life course. Human stress genomics considers the role of individual stress perceptions on gene expression. Yet, the role of structurally rooted biopsychosocial processes that may be activated by the social patterning of stressors in an historically unequal society, whether perceived by individual black women or not, could also impact cellular physiology and gene expression patterns relevant to HR- breast cancer etiology. Using the weathering hypothesis as our conceptual framework, we develop a structural perspective for examining racial disparities in breast cancer subtypes, integrating important findings from the stress biology, breast cancer epidemiology, and health disparities literatures. After integrating key findings from these largely independent literatures, we develop a theoretically and empirically guided framework for assessing potential multilevel factors relevant to the development of HR- breast cancer disproportionately among Black women in the US. We hypothesize that a dynamic interplay among socially patterned psychosocial stressors, physiological & behavioral responses, and genomic pathways contribute to the increased risk of HR- breast cancer among Black women. This work provides a basis for exploring potential alternative pathways linking the lived experience of race to the risk of HR- breast cancer, and suggests new avenues for research and public health action.

## 1. Introduction

Breast cancer is widely recognized as a highly heterogeneous disease, commonly characterized by the gene or hormone receptor expression pattern of the tumor [1]–[5]. While racial disparities across the continuum of breast cancer care are well-documented, differences in the distribution of breast cancer subtypes among White and Black women have garnered a significant amount of attention [6]–[9]. Numerous population-based studies have found that, compared to White women with breast cancer, Black women are approximately twice as likely to be diagnosed with estrogen receptor negative (ER-), estrogen and progesterone receptor negative (ER-/PR-), or triple negative tumors (ER-, PR- and human epidermal growth factor receptor, HER2 negative), subtypes of the disease [10]–[17]. This statistically significant disparity has meaningful clinical implications, as hormone receptor negative (HR-) tumors are associated with larger and higher-grade carcinomas at the time of diagnosis and are not responsive to current endocrine-based treatments such as Tamoxifen and Herceptin. As a result, women diagnosed with HR- tumors have higher rates of five-year cancer-related mortality than women diagnosed with other types of breast cancer, regardless of tumor stage at the time of diagnosis [15]. Moreover, as breast cancer subtype is thought to be determined at the onset of tumor development, observed differences in subtype across racial groups are less likely to be influenced by access to breast cancer screening, diagnostic, and treatment resources [18]. As a result, identifying factors that influence the development of HR- breast cancer may be critical to developing upstream interventions to reduce mortality disparities.

The disproportionate incidence of biologically distinct breast cancer subtypes across racial groups has led to the investigation of potential genetic risk factors that are associated with African ancestry, and therefore may place Black women at a higher risk for HR- breast cancer [19]. Part of the rational for this line of research has been that HR- breast cancers are also more commonly diagnosed among carriers of mutations in the *BRCA1* gene, providing some evidence for germline genetic risk factors in the development of specific breast cancer subtypes [20]. BRCA1 founder mutations have been well characterized in the Ashkenazi Jewish population, but similarly prevalent and pathogenic founder mutations have not yet been identified among Black women [21]. There is also no evidence that Black women have a higher population prevalence of *BRCA1* mutations than non-Ashkenazi White women, although the sample size within the available studies is limited [9], [22].

The emphasis on heritable risk factors fails to consider the potential effects of the acquired biological changes that may result from differential exposure to racially-stratified social and physical environments over the life course. The emerging field of human social genomics takes an important step in this direction, demonstrating that social conditions can influence gene expression and that our molecular make-up is mutable according to social factors [23]. However, human social genomics emphasizes one important aspect of social conditions – the individual's subjective perceptions of them – and focuses on this individual psychological approach. By studying the neural and molecular mechanisms that mediate the effects of social processes on gene expression, and the genetic polymorphisms that moderate individual differences in genomic sensitivity to social context, the goal of human social genomics research is to produce molecular models of how social and genetic factors interact to shape complex behavioral phenotypes and susceptibility to disease.

Our reading of the population health disparities literature leads us to apply a more comprehensive model for thinking about the role of social conditions in the production of the racial patterning of breast cancer subtype in the US, the weathering hypothesis [24]–[27]. Weathering is a cumulative stress perspective grounded in social research that draws on stress physiology to posit that prolonged psychosocial or physical challenges to metabolic homeostasisin socially marginalized groups - whether objective or subjective – increases the risk of disease [28], leads to early onset of chronic disease [29], and accelerates cellular aging [30] across the young adult through middle ages. In this conceptual model, the health implications of race are contingent, context dependent, fluid and cumulative. Going beyond the individual stress perceptions critical to human stress genomics, the weathering approach raises the question of whether it might also be promising to consider how molecular mechanisms – including those that affect breast cancer subtype – may be activated by the broader social patterning of stressors in a race-conscious and unequal society. For example, social policies that disrupt collective coping approaches such as the strategies for overcoming material hardship on which members of marginalized groups depend, can undermine the social ties and social support that are protective against stressors [31]. Thus, the weathering conceptual model extends human stress genomics to consider not only individual perceptions, but also the physiological implications of historically structured differences by race in lived experience, exposure to stressors, and access to coping resources over the life course [32].

As suggested in a recent review [33], the weathering hypothesis is especially relevant in the case of racial disparities in breast cancer subtype due to both the population at highest risk and the conceptual and methodological gaps in the existing literature.

First, premenopausal Black women are at particularly high risk both for experiencing weathering processes, and for developing HR- and triple negative tumors [34]–[37]. Indeed, the most consistently significant factors associated with increased risk of aggressive breast cancer subtypes have been Black race and younger age of breast cancer onset [10], [15], [34]–[39]. Analyses of subtype-specific breast cancer risk factors are typically adjusted for race, age, and the crude measures of socioeconomic status available in cancer registry data files, without further consideration of interrelated contextual factors.

Second, rather than rely on the main effects of conventional, area-based socioeconomic variables alone as the measures of social conditions, or viewing them as individual characteristics, the weathering approach recognizes the importance of considering impacts of interactions among socioeconomic indicators and race. Moreover, the weathering approach considers the impacts of lived experiences for whole communities, social identity groups, and populations. In turn, a broader universe of hypotheses for explaining racial patterning of breast cancer subtype in the US can emerge, along with clearer explication and deeper understanding of the multiple structural, psychosocial, and biobehavioral pathways that may contribute to the observed racial disparities in breast cancer subtype.

To this end, we have applied the weathering hypothesis to develop a new conceptual model for breast cancer subtype disparities research. We begin by critically reviewing the literature on stress and breast cancer risk. Next, we present recent evidence suggestive of the potential importance of structural and neighborhood factors in risk of aggressive breast cancer subtypes, including new evidence from the California Cancer Registry. We then describe our conceptual model, identifying key factors across multiple levels that should be included in future breast cancer subtype disparities research, and conclude with specific suggestions for future model-guided research.

## 2. Materials and Methods

To develop a more integrative approach to assessing social, behavioral, and genomic factors that may contribute to breast cancer subtype disparities, we critically reviewed and summarized papers from the stress biology, breast cancer epidemiology, and health disparity literatures that were abstracted in PubMed and/or Web of Science prior to June 1, 2017. As described in the Introduction, we used the weathering hypothesis as a theoretical basis for integration of these distinct literatures. We then developed a new, multilevel conceptual model for examining racial disparities in breast cancer subtypes based on the peer-reviewed manuscripts germane to the constructs of interest: racially patterned structural, residential, and individual exposures that are theoretically or empirically linked to ER- breast cancer risk factors. We present preliminary findings informed by this model that are suggestive of the role of social structural factors on breast cancer subtype. Finally, we describe two transcriptional regulation pathways which provide a plausible route for these multilevel exposures to “get under the skin” and contribute to the development of ER- breast cancer. By considering the social, behavioral, and biological factors elucidated in our conceptual model, multi-pronged interventions may be developed [40], [41].

For the purposes of this paper, “Black” refers to individuals who self-identify with this loosely defined, highly heterogenous racial/ethnic group. Our discussion of race will center on the social construction of majority and minority groups within the American culture, and in no way implies a biological basis for this stratification.

This literature review did not meet the definition of human subject research and thus was exempt from Institutional Review Board oversight.

## 3. Results and Discussion

### 3.1. Review of evidence: Stress as a breast cancer risk factor

The theoretical and empirical relationship between stress and various health outcomes has been well documented, and the investigation of stress as a risk factor for breast cancer is also not a new proposition [42]–[44]. Prior studies investigating the potential link between various types of psychosocial stressors and breast cancer have produced mixed or null findings, but they have almost uniformly suffered from significant methodological issues that limit the strength of their interpretations (Table 1). Two reviews and meta-analyses of this literature shed light on these limitations. Petticrew and colleagues identified 29 studies conducted between 1966 and 1997 that met their inclusion criteria, yet only one was prospective, the gold standard for evaluating the impact of a potential risk factor that could be strongly influenced by recall bias or the development of the disease [45]. Chida *et al*. [46] completed a meta-analysis of 83 prospective community-based breast cancer studies that examined associations between stress-related psychosocial factors and cancer incidence, survival, and mortality. While no association was seen between the individual-level psychosocial factors measured (*e.g.*, stressors, poor social support, or poor quality of life) and community-based breast cancer incidence or mortality, they noted a significant negative relationship with breast cancer-specific survival (combined hazard ration 1.13, 95%CI: 1.05-1.21).

Some of the variation in results may be attributed to limitations of stress assessments. Many studies included in the two meta-analyses used simple stress checklists such as the Social Readjustment Rating Scale [47]. The use of such checklists presents measurement issues such as lack of event severity ratings and other contextual information about the events and the respondent. This information is critical, because being exposed to a stressor may neither elicit distress nor the same degree of distress in every individual. Individual-level response to some stressors may depend on multiple exogenous factors such as emotional resiliency, socioeconomic position, or the type and amount of available social support [48], [49]. The basic stress checklist approach fails to consider whether multiple events are interrelated (*i.e.*, going through a divorce and change in financial state) or multiplicative in their effects, rather than simply additive. Similarly, some items included on the standard stress checklists could occur concurrently or following a breast cancer diagnosis, making positive associations uninterpretable without additional contextual or temporal information. Studies included in the meta-analyses also varied widely in their stress measurement timeframe, with some studies assessing only stressful events within the past year, whereas other studies measured stress over the participants' lifetime.

**Table 1.** Summary of papers illustrative of current gaps and future directions for stress and breast cancer subtype research.

**Ref.**	**Type of study**	**Main finding**	**Study strengths and limitations**
Petticrew *et al*. [45]	Meta-analysis; 29 breast cancer studies	No significant association between breast cancer and either bereavement or adverse life events	Strength: Assessed study quality and potential for publication biasLimitations: Narrow definition of stress and stressors; almost entirely retrospective data; no differentiation between breast cancer subtypes
Chida *et al*. [46]	Meta-analysis; 83 breast cancer studies	Significant association between stress and breast cancer survival, but not incidence	Strength: Included more recent studies than Petticrew, *et al.*Limitations: Very wide definition of stress and stressors, some of which may not be equivalent as assumed when generating meta statistics; no differentiation between breast cancer subtypes
Cheang and Cooper [47]	Retrospective study; recall of stressful life events in 2 yr preceding breast biopsy (*n*=121) or while attending a well women clinic (*n*=42)	Women diagnosed with breast cancer reported more stressful life events than women with normal biopsies or healthy controls	Strength: Assessment of stressful life events, while still retrospective, was conducted prior to the diagnosis of breast cancerLimitation: Narrow definition of stress and stressors; analysis did not adjust for potentially confounding age differences between groups; no differentiation between breast cancer subtypes
Spiegel *et al*. [53]	RCT; intensive group therapy *vs* educational materials among women with metastatic breast cancer	Women with ER- breast cancer randomized to intervention had significantly longer mean survival time than ER- women in the control arm; no significant differences in survival among ER+ women	Strength: Randomized control design; outcomes assessed by breast cancer subtypeLimitation: Did not directly measure stressors prior to diagnosis or following the intervention
Michael *et al*. [54]	Prospective cohort study; life events, social support, and breast cancer incidence among the Women's Health Initiative observational study participants	A small but significant increased risk of breast cancer among Black women reporting 1 “severely stressful” life event	Strength: Large, prospective, well-documented cohort with substantial data on other breast cancer risk factorsLimitation: Relatively small number of Black women; limited to post-menopausal breast cancer incidence; no differentiation between breast cancer subtypes
McClintock *et al*. [57]	Animal model; social isolation conditions and development of mammary tumors in Sprague-Dawley rats	Socially-isolated rats developed mammary tumors at a significantly higher rate at an earlier age than their group-housed litter mates	Strength: Well-controlled study using a socially-oriented model animal; observed tumors are clinically similar to aggressive human breast cancer subtypesLimitation: Translation to human social and biological processes is needed
Williams *et al*. [58]	Animal model; social isolation conditions and development of mammary tumors in Tag transgenic mice	Socially-isolated mice developed larger mammary tumors at a faster rate than their group-housed litter mates	Strength: Well-controlled study replicating the findings of McClintock *et al*., in a different socially-oriented species; observed tumors are clinically similar to aggressive human breast cancer subtypes Limitation: Translation to human social and biological processes is needed
Hasen *et al*.[59]	Animal model; social isolation conditions and development of mammary tumors in p53 knockout mice	Socially-isolated mice developed fewer of mammary tumors than their group-housed litter mates	Strength: Well-controlled study using a common mouse model for cancer studies Limitation: p53 knockout mice are genetically susceptible to multiple types of tumors and may not be an appropriate model for social influences on mammary tumor development
Taylor *et al*. [61]	Prospective cohort study; racial discrimination and breast cancer incidence among Black Women's Health Study participants	Women under the age of 50 who reported either major discrimination in the workplace, or across three domains (workplace, housing, and police) had significantly higher odds of being diagnosed with breast cancer during the follow-up period. No significant relationships between racial discrimination and breast cancer incidence among Black women over age 50.	Strength: Large prospective cohort of Black women with appropriate adjustment for other breast cancer risk factors Limitation: No assessment of coping or other sources of mitigating or exacerbating factors; Study population is not necessarily representative of the US Black population (*e.g.*, higher levels of education and SES)
Krieger *et al*.l [62]	Case-only study; Jim Crow state of birth and ER status among women diagnosed with breast cancer in a SEER-13 catchment area	Black women with breast cancer who were born in a Jim Crow state had significantly higher odds of ER- subtype relative to Black women born in other states. No association between state of birth and odds of ER- *vs* ER+ subtype among White women.	Strengths: Use of data from a nationally representative collection of cancer registries; takes a structural approach Limitations: Small number of women who were born after 1965 and diagnosed with breast cancer during the study period limits the statistical power for comparison across both geography and historical period
Barrett *et al*. [66]	Case-only study; neighborhood SES, neighborhood SES change (gentrification), and odds of distant metastasis at time of breast cancer diagnosis among Cook County, IL women	Both concentrated neighborhood disadvantage and upward neighborhood socioeconomic change were associated with increased odds of distant metastasis at time of diagnosis.	Strengths: Contextualized the neighborhood socioeconomic environment beyond static measures of advantage or disadvantageLimitations: Relevant individual-level data, including SES and length of residence within the neighborhood, were not available in the cancer registry data file; breast cancer subtype was not reported
Warner and Gomez [68]	Case-only study; racial residential segregation, neighborhood racial concentration, and odds of late-stage diagnosis, breast cancer-specific mortality, and all-cause mortality among California women	Within more segregated metropolitan regions of California, Black women with breast cancer who lived in neighborhoods with lower percentages of Black residents had higher odds of late-stage diagnosis, and higher hazard ratios for breast cancer-specific & all-cause mortality	Strengths: Accounted for both regional patterns of residential segregation (potential proxy for broader racially stratified policies and opportunities) and neighborhood-level racial concentration (potential proxy for available social ties); adjusted for other relevant clinical featuresLimitations: Relevant individual-level data, including SES and length of residence within the neighborhood, were not available in the cancer registry data file
Linnenbringer [69]	Case-only study; racial residential segregation, neighborhood racial concentration and SES, and odds of breast cancer subtypes among California women	Within more segregated metropolitan regions of California, Black women with breast cancer who lived in neighborhoods with lower percentages of Black residents had higher odds of HR- breast cancer	Strengths: Extended work of Warner and Gomez to breast cancer subtypes as the outcome of interest; adjusted for other relevant clinical featuresLimitations: Relevant individual-level data, including SES and length of residence within the neighborhood, were not available in the cancer registry data file

RCT: Randomized clinical trial; ER: Estrogen receptor; HR: Hormone receptor; -: Negative; +: Positive; SES: Socioeconomic status.

Another major methodological limitation we observed is the lack of adjustment for known breast cancer risk factors. For example, Cheang and Cooper's [50] limited-prospective study found that the women who were diagnosed with breast cancer report significantly more stressful life events and life events than the women who were diagnosed with benign breast disease or healthy controls. However, they did not adjust for potential confounders nor baseline demographic variation. Of note, the cases in this study were, on average, 2.5 yr older than the women in the benign breast disease group and 7.5 yr older than the healthy controls. Given the age distribution of breast cancer diagnoses is bimodal with peaks at age 50 (predominately HR-negative breast cancer) and age 70 (predominantly HR-positive breast cancer) [1], it is difficult to assess whether increasing age or increasing number of life events were most salient to breast cancer risk.

While more recent studies have taken into account other breast cancer risk factors, virtually all of the existing stress-related research studies have treated breast cancer risk as a single, uniform entity. With the establishment of breast cancer subtypes, it has become quite clear that breast cancer is a heterogeneous set of conditions with distinct risk factors, etiologies, molecular signatures, and natural histories [51]. This heterogeneity has largely been unaccounted for in the stress and breast cancer risk literature, as none of the studies in the four meta analyses described above stratified their cases by breast cancer subtype. This lack of subtype specificity may be a major contributor to the largely equivocal results, as the effects of stress on breast cancer subtypes may very well be different given the known effects of stress on the endocrine system. For example, chronic psychosocial stress can lead to disruption of the hypothalamic-pituitary-gonadal (HPG) axis, which in turn lowers the level of endogenous estrogen production [52]. As a result, risk for ER-positive tumors could actually be reduced among individuals exposed to chronic stress, while ER-negative tumor risk may be unaffected or even increased *via* other stress-related neuroendocrine or telomere length pathways.

One randomized trial of an intensive group therapy intervention among women diagnosed with metastatic breast cancer did stratify the results by ER status, and provides the first empirical justification for stratifying by breast cancer subtype. Spiegel and colleagues [53] found that the ER-negative women randomized to the experimental arm survived a median of 29 mo compared to only 3 mo in the control group, who received only educational materials. There was no significant difference in survival between ER-positive women randomized to the intervention or the control arm. While the intervention did not measure stress levels directly, the findings imply that reducing stress *via* intensive therapy has greater survival benefits for women with a more aggressive breast cancer subtype. This finding supports the hypothesis put forth by Chida, *et al.* (2008) in that there may be several direct physiological pathways that may link psychosocial stress to cancer survival, including: impaired DNA repair mechanisms, promotion of tumor migration and infiltration *via* changes in glucose uptake rates, and increased tumor vascularization.

Of note, a more recent analysis of stress and breast cancer among participants in the Women's Health Initiative found that increased stress was associated with lower risk of post-menopausal breast cancer [54]. However, reports of one “severely stressful life event” were associated with a small (but statistically insignificant) increase in breast cancer risk only among Black women. Melhem-Bertrandt and Conzen [55] suggest that the theorized differential effects of stress on breast cancer subtype should be considered in addition to the “underlying population-based differences” in subtype risk (p. 133). Perhaps a better question may be whether population-level differences in breast cancer subtype reflect population-level differences in exposure to – and physiological consequences of – chronic and severe stress.

### 3.2. Consideration of specific social stressors: Social isolation and racial discrimination

More recent research regarding stress and breast cancer has focused on the biological and/or epidemiological role of two types of social stressors: perceived social isolation and racial discrimination. Perceived social isolation has been repeatedly attributed to increased risk of morbidity and mortality, although the precise mechanisms by which social isolation impacts health remain unclear [56]. A murine model study by McClintock *et al.* [57] suggests that social isolation could be associated with the development of breast cancer that is clinically similar to HR- breast cancer in humans. In this study, genetically identical female Sprague-Dawley rats – who naturally engage in social behaviors such as co-rearing pups – were randomized to either normal group housing or socially isolated cages. All food and exercise conditions were held constant. Yet, the socially-isolated rats developed mammary carcinomas at a significantly higher rate at an earlier age than their group-housed counterparts. Williams *et al.* [58] also found that female Tag transgenic mice suffered from increased rates of mammary tumor growth and tumor size when subjected to social isolation. However, others have reported that a different breed of socially-isolated mice actually had lower numbers of mammary tumors than their group-housed counterparts [59]. Melhem-Bertrandt and Conzen [55] posit that this was due the use of a p53 knockout mouse model, which has a fundamentally different source of genetic susceptibility to mammary tumors. Determining whether a similar phenomenon occurs in human populations, particularly among Black or other disadvantaged groups, is an important public health question.

Research regarding the potential relationships among subtype-specific breast cancer risk and racial discrimination is also limited but growing. At the individual level, perceived discrimination has been implicated in poor physical and mental health among minorities [60] and with increased risk for breast cancer among Black women under the age of 50 [61]. Although breast cancer subtype was not directly assessed, HR- breast cancers are more common among premenopausal Black women than any other demographic group [38].

The work of Taylor *et al.* [61] further supports the hypothesis that race-related stress experienced over the life course may affect breast cancer risk. In their study of over 49,000 Black Women's Health Study participants, they found that women under the age of 50 who reported major discrimination in the workplace had an adjusted breast cancer incidence rate ratio of 1.32 relative to women in the same age group who did not report workplace discrimination (95%CI: 1.03-1.70). In addition, women under age 50 who reported three domains of major discrimination (workplace, housing, and by police) had a 1.48 adjusted incidence rate ratio relative to women who had not experienced discrimination in these areas. That similar relationships were not seen among women ages 50 or older further supports the notion that stress across the lifespan may affect reproductive hormone expression. Hormone receptor status was not reported for the 593 self-reported breast cancer cases, however.

More recent work found that Black women with breast cancer who were born in a Jim Crow state – that is, one in which racial discrimination was legally codified prior to the US Civil Rights Act of 1964 – had significantly higher odds of ER- subtype relative to Black women born in other states, but there was no such geographic difference in breast cancer subtype odds ratios among White women [62]. While individual-level measures of racial discrimination are not available through the SEER cancer registries on which this analysis was based, and possible mechanisms were not explored, these findings suggest that exposure to structural racism is associated with odds of aggressive breast subtypes. The potential impacts of structural racism have not been widely considered in the literature on black/white differences in breast cancer, yet we argue that they should be.

### 3.3. Rationale for an alternative conceptual model

**Figure 1.**
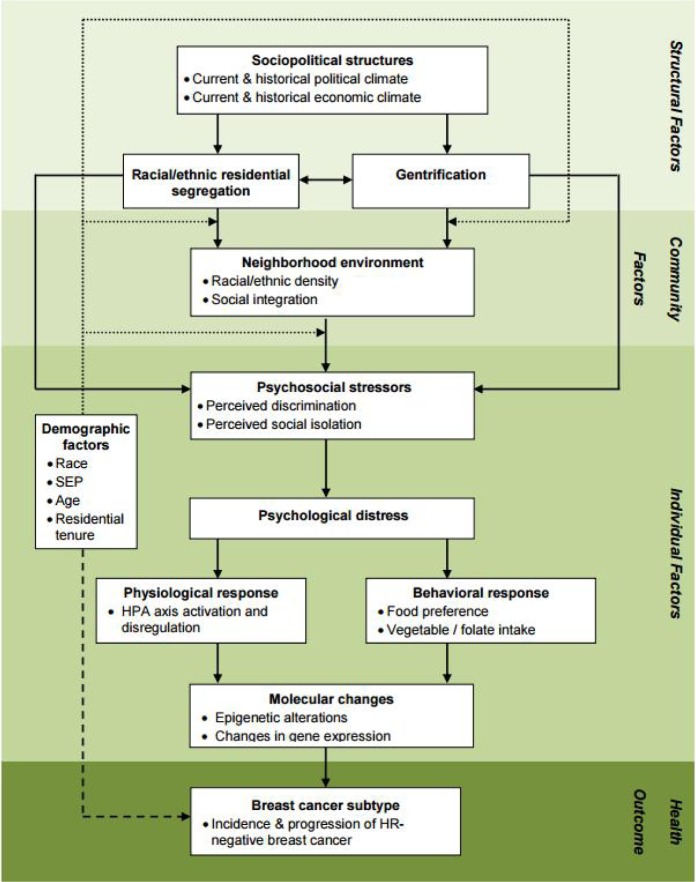
Conceptual model of the relationship between stress and HR-negative breast cancer. Solid arrows: direct (box to box) or moderating (box to arrow) relationships; heavy dashed arrow: direct association between race and breast cancer subtype typically reported in the literature; dotted arrows: alternative avenues by which sociodemographic factors may interact with key constructs.

Based on the literature reviewed thus far, an intriguing portrait of racial disparities in breast cancer subtype emerges. Relative to Whites, Black women are approximately 2 to 3 times more likely to develop HR- breast cancer. This subtype is clinically, epidemiologically, and molecularly distinct from the most common, hormone receptor positive (HR+) form of breast cancer. These subtype distinctions have not been accounted for in the majority of prior research regarding the relationship between stress and breast cancer incidence and mortality. Similarly, racial differences in the exposure to stressors and the availability of coping resources have not been accounted for in much of the existing breast cancer health disparity research. In fact, a large portion of breast cancer disparity research has focused upon possible genetic risk factors associated with African ancestry. Rather than continuing this simplistic search for risk factors in Black women's genotype, we propose an alternative model that explores the implications of the phenotype of being Black in America, particularly regarding exposure to structurally rooted chronic stressors and strains (Figure 1). In the following sections, we introduce structural- and community-level factors that may serve as important sources of racial variation in exposure to key stressors and coping resources. The remainder of the model and this paper provides a general overview of potential behavioral and biological pathways that may connect structurally patterned stress to the incidence and progression of HR- breast cancer.

### 3.4. Structural-level factors

Race-based residential segregation is a potential form of structural inequality that may be associated with the uneven distribution of breast cancer subtype. The unequal distribution of material, psychosocial, and other resources across segregated neighborhoods contribute to racial disparities in many aspects of American life, including socioeconomic position (SEP) and health [63]–[65]. As a result, evaluations of health disparities such as those seen in breast cancer subtype should consider what roles residential segregation and its implications for black women's SEP might have in the creation or propagation of observed racial difference in health outcomes.

Previous studies of disparities in breast cancer subtype have largely failed to consider structural factors such as residential segregation. However, a small number of recent studies have begun to elucidate how these factors relate to observed racial inequalities in breast cancer subtype. They suggest the value of considering collective social constructs and also interactions rather than only main effects of social variables. Barrett *et al.* [66] examined associations between presence of a distant metastasis at diagnosis and neighborhood characteristics of concentrated disadvantage, concentrated affluence, and upward socioeconomic change among women diagnosed with breast cancer in Cook County, Illinois between 1994 and 2000. Women's home address at the time of diagnosis was geocoded to the census tract level, which served as the community-level unit of analysis. Census-based measures of concentrated disadvantage and concentrated affluence were created [67]. A composite measure comparing 1990 and 2000 U.S. Census data on the value of owner-occupied housing, percent of civilian labor force employed in professional or managerial roles, and the percent of college-educated adults within a census tract was used to create an upward socioeconomic change score. A multilevel logistic regression analysis identified concentrated affluence to be inversely related to distant metastasis at diagnosis (OR = 0.86; 95%CI: 0.79-0.93) while both concentrated disadvantage (OR = 1.23; 95%CI: 1.12-1.36) and upward socioeconomic change (OR = 1.09; 95%CI: 1.01-1.18) were directly associated with increased risk of distant metastasis at diagnosis. While the benefits for health of concentrated affluence and the disadvantages of concentrated poverty are intuitive, the finding that upward socioeconomic change in one's residential area is directly associated with the risk of distant metastasis at diagnosis is particularly intriguing from a weathering perspective. Rather than reify the presumed benefits of affluence for anybody in any context, it provides an example of an important interaction and signals the need to consider the effects of socioeconomic variables on health to be contextually fluctuating. The original residents' experience of upward socioeconomic change may be contingent upon its implications for their social ties, sources of social support, and identity affirmation. In some contexts, these implications may, on balance, be negative.

Warner and Gomez [68] examined relationships between Black-White residential segregation and stage at breast cancer diagnosis, breast cancer-specific and all-cause mortality in California between 1996 and 2004 using available data. Compared to residents of low segregated regions, Black women living in neighborhoods with low percentages of Blacks within highly segregated regions had higher odds of being diagnosed with distant-stage cancer (OR = 2.11; 95%CI: 1.05-4.27). Black women diagnosed with breast cancer had lower levels of breast cancer specific (HR = 0.86; 95%CI: 0.76-0.97) and all-cause mortality (HR = 0.90; 95%CI: 0.82-0.99) in neighborhoods with at least 20% Black residents. While ER and PR status were available on approximately 70% of the included breast cancer cases, this information was only used to describe the overall study population and make statistical adjustments in the survival models.

Building on the Warner and Gomez analysis, Linnenbringer and colleagues used California Cancer Registry data geocoded and linked to Census block group sociodemographic characteristics and metropolitan-level measures of racial segregation to examine how neighborhood-level socioeconomic status, neighborhood-level racial concentration, and metropolitan level racial segregation may relate to the odds of having HR- breast cancer, relative to the HR+ subtype [69], [70]. Adjusting for individual-level sociodemographic (age, marital status, and insurance status) and clinical characteristics (tumor stage and grade), and neighborhood median household income, we found that Black women diagnosed with breast cancer between 1996-2004 had a statistically-significant 2.7% lower odds of HR- subtype with every 10% increase in the proportion of Black neighborhood residents. While the observed reduction in odds of HR- breast cancer relative to HR+ breast cancer was evident in the full sample of Black women with breast cancer, stratification by metropolitan-level racial segregation levels show the relationship was statistically significant only among Black women living in the metropolitan statistical areas (MSA) that were in highest tertile of California's 25 MSA's, based on entropy, a 2000 Census-derived measure of racial segregation. We hypothesize that, within highly-segregated metropolitan areas, the psychosocial and physiological effects of reduced exposure to racial discrimination and/or increased availability of social support among Black women residing among more Black peers could influence subtype-specific breast cancer risk.

The above three studies suggest that gentrification, residential segregation and racial discrimination are associated with stage at breast cancer diagnosis, cancer-specific mortality and all-cause mortality, and breast cancer incidence, respectively. In this context, we highlight the findings that might, at first blush, appear counterintuitive: ➀ that living in neighborhoods that experienced positive economic change was directly associated with an increased odds of being diagnosed with distant-stage cancer among black women; and ➁ that living in a hypersegregated neighborhood was associated with decreased odds of HR- breast cancer subtype among black women. In addition to supporting a view of breast cancer subtype as mutable according to broad social conditions, rather than programmed in ancestral DNA, these findings suggest that larger structural issues may work directly or through what happens in neighborhoods and communities to initiate or mitigate collective distress and activate harmful physiological mechanisms.

### 3.5. Neighborhood- and community-level mechanisms linking structural factors to breast cancer subtype

Several neighborhood- and community-level factors might serve as intermediaries between structural factors and psychosocial, behavioral, and biological factors that increase a woman's risk of developing HR- breast cancer. We define neighborhoods both in terms of the people and the institutions within a geographic area, both of which are influenced by the structural and cultural forces of the larger ecological systems (*e.g.*, cities, states, nations) in which they are nested [71]. The association between neighborhood and health reflects a dynamic interaction between the characteristics of those who live in a neighborhood and the characteristics and resources of the neighborhood [72]–[74]. From this vantage point, race-based residential segregation and the underlying sociopolitical structures that support it are salient. Likewise, both the social and physical environment each affect the stressors and psychosocial buffers present within a neighborhood.

While race-based residential segregation negatively impacts minority residents in several health-relevant domains, it may also provide some limited advantages. Predominantly minority neighborhoods may be economically and politically marginalized, but they may offer other health benefits that derive from shared alternative cultural frameworks, deeply rooted social ties, and organized networks across families who pool economic risk to protect against severe material hardship of individual families. These attributes of segregated neighborhoods shape the experiences of residents [49], [75]–[77], which may in turn protect against the worst race-related stressors and contribute to social integration [78]. It is important to note that long-term residents of neighborhoods experiencing upward socioeconomic change may view the neighborhood differently from others [79], [80]. In a recent study of a redeveloped, mixed income housing development, longer-term residents had qualitatively different appraisals of their neighborhood than newcomers [80]. These appraisals had a significant impact on their level of neighborhood engagement, and ostensibly their experience of social integration.

Social integration may have an indirect effect on breast cancer subtype *via* chronic exposure to psychosocial stressors and subsequent physiological and behavioral stress responses. Barrett *et al.* [66] provide some of the first empirically-based theoretical evidence for a relationship between neighborhood social networks and breast cancer disparities. The authors hypothesize that changes in neighborhood levels of social integration may contribute to the observed association between upward socioeconomic change and distant metastasis at diagnosis. This hypothesis implies that long-time Black residents who remain in rapidly gentrified neighborhoods may suffer worse breast cancer outcomes, due in part to the decreased social integration of the neighborhood. While the authors did report that Black women also had a greater chance of having a distant metastasis at diagnosis (OR = 1.24; 95%CI: 1.03-1.48), they neither discussed the results of their neighborhood-level findings in terms of potential confounders with race due to race-based residential segregation, nor discussed whether Whites and Blacks might be equally affected by the neighborhood conditions measured. Further investigation of potential interactions among race, upward neighborhood socioeconomic change, and social integration within the context of breast cancer subtype is needed.

### 3.6. Individual-level factors mediating the relationship between community-level factors and breast cancer subtype

#### 3.6.1. Psychological distress

According to several models, distress is an important mediator between structural factors (*e.g.*, neighborhood disinvestment), individual-level factors (*e.g.*, perceived social isolation) and health outcomes [43], [81], [82]. The relationship between exposure to individual-level stressors and resultant distress is proximal to the physiological and behavioral responses that may influence cellular changes associated with breast cancer subtype. This distinction is important because, as discussed earlier, simply being exposed to various community-level factors or psychosocial stressors may not necessarily generate distress resulting in the physiological or behavioral responses described below.

Several authors have suggested that exposure to stressors associated with social disadvantage increases vulnerability to mental and physical health problems [27], [83]. Psychosocial stressors may trigger biophysical responses leading to increased risk of HR- breast cancer. In addition, as has been suggested by Jackson and colleagues, exposure to psychosocial stressors may lead individuals to engage in health behaviors that, while quelling distress in the short-term, also lead to biophysical pathways related to development of HR- breast cancer [84].

A recent report provides evidence that individuals with high self-reported levels of social isolation express genes that lead to over-activation of genes involved in the inflammatory response system, and under-activation of glucocorticoid response elements that are critical to the anti-inflammatory response system [85]. Cole puts forth a helpful illustration of the potential pathways, depicting a dynamic flow of information from the social environment to protein formation, health, and behaviors *via* perceptions formed in the central nervous system, neuroendocrine responses, and transcriptional regulation of gene expression. This framework holds promise for exploring how exposure to social stressors more prevalent among Black women result in increased incidence of aggressive breast cancer subtypes.

#### 3.6.2. Physiological responses

Distress activates the hypothalamic-pituitary-adrenal (HPA) axis. This feedback system prepares the body for responses to stressful situations, such as signaling for increased cortisol secretion, in order to utilize stored energy and respond to threats [86]. Yet, the inability to efficiently turn off the HPA axis following chronic exposure to stress – commonly referred to as allostatic load – has been associated with dysregulation of glucocorticosteriods, neurotransmitters, and inflammatory cytokines [87]. Persistent activation has detrimental effects on existing cellular systems, including dysregulation and acceleration of normal cellular aging process [88]. Having a high allostatic load has been construed as an indicator of weathering and age-patterns of allostatic load in young through middle adulthood have been found to be higher and steeper for Black compared to White women in the US [28]. While the relationship between allostatic load and breast cancer risk has not been prospectively measured, a recent analysis of National Health and Nutrition Examination Survey data found that having a personal history of breast cancer was associated with higher levels of allostatic load among Black women, but not among White women [89]. Further study is needed to determine the directionality of this relationship.

#### 3.6.3. Behavioral response

Dietary behaviors represent a potentially important mediator on the pathway from neighborhood- and community-level factors to breast cancer-related molecular changes. For example, neighborhoods with a high percentage of minority residents are less likely to have chain supermarkets located nearby [90]. As a result, residents of these neighborhoods tend have limited access to fresh fruits and vegetables [91], [92]. The combination of restricted availability of healthful foods with the pervasive presence of less healthful fast foods has a significant impact on dietary behaviors [93]. In addition to the direct relationship between material resources and dietary behaviors, eating comfort foods, which are typically high in fat and/or sugar, may be an individual-level response to distress that actually helps dampen the stress response system that is activated *via* the HPA axis [84], [94].

One potential implication is that Black women in lower-resourced neighborhoods who are exposed to significant amounts of stress may not get enough folate, which is found in green leafy vegetables and fruits, has an important role in the maintenance of proper DNA methylation patterns [56]. Women who consume less folate are more likely to be diagnosed with estrogen receptor negative tumors [95]. The Black Women's Health Study found that total vegetable intake was inversely associated with risk of ER-negative / PR-negative breast cancer, even after adjusting for 15 other known or suspected breast cancer risk factors, such as use of hormone replacement therapy [96]. The authors also reported a trend, albeit statistically insignificant, towards a similar inverse relationship between cruciferous vegetables and ER-negative / PR-negative breast cancer. That no significant relationship was found between ER-positive breast cancers and vegetable intake suggests variation in the etiology and risk factors for breast cancer subtypes. Whether a similar relationship exists between HR- breast cancers and vegetable and/or folate consumption remains to be determined.

#### 3.6.4. Molecular changes

The gene-environment interactions most relevant to the development of aggressive breast cancer subtypes may occur at the transcriptional level, due to changes in DNA methylation patterns or other complex molecular pathways implicated in human social genomics. DNA methylation occurs when a group of molecules attach methyl groups to the specific areas of a gene's promoter region, thereby preventing the “reading” of the gene and the formation of the gene product. DNA methylation (and de-methylation) is a generally stable set of processes that can be replicated from parent cell to daughter cell. However, an individual's DNA methylation patterns may also change over time, including in response to social and environmental factors.

Disruptions in the DNA methylation process are thought to be especially important in the development and proliferation of cancerous cells [97], [98]. For cancerous cells to continue to grow and divide at a rapid pace, tumor suppressor genes need to be silenced *via* a deleterious gene mutation or gene-specific hypermethylation. Two recent studies suggest that as cells age, chromosome instability increases and hypermethylation of tumor suppressor genes is more prevalent [99], [100]. Additionally, tumor enhancing genes (*i.e.*, oncogenes) must be activated *via* general hypomethylation. Although the exact mechanisms that cause gene-specific hypermethylation and general hypomethylation in cancerous cells are not well-characterized. However, evidence is growing to indicate that cellular aging, as well as elements of the physical and social environment, play a role in this process [101].

Evidence for the relationship between cellular aging and hypermethylation comes from a monozygotic twin study [102]. In this study, monozygotic twins who were less than 28 years old, and particularly those who were still in early childhood, exhibited very similar DNA methylation patterns. However, sets of twins older than 28, especially those who were middle aged and older, were found to have significantly different DNA methylation patterns across their genome. Whether the evolution of an individual's DNA methylation pattern is the result of more typical cellular aging processes or repeated environmental and/or psychosocial insults that are part of the weathering process has yet to be determined.

As noted by Javonovic *et al.* [103], the primary epigenetic mechanism of interest with regard to estrogen receptor expression status has been DNA hypermethylation of the estrogen receptor alpha (*ER-α*) gene promoter region, ESR1. This is intuitive, because increased methylation of a promoter region results in the down regulation or silencing of gene's expression, which would thereby explain the lack of estrogen receptors in an ER-negative tumor. Indeed, in vitro laboratory work in the mid-1990's supports this developmental pathway for ER-negative tumors. However, subsequent clinical studies have produced conflicting results[104], [105]. In one study, 76% of ER-negative breast cancers were found to have a methylated *ER-α* gene, while 22% of ER-positive tumors also demonstrated methylation of the *ER-α* gene [106]. This suggests that selective methylation of the *ER-α* gene plays an important, yet insufficiently understood, role in the development of HR- breast cancer. While Gaudet *et al.* [107] found no clear association between promoter methylation levels and *ER-α* expression levels, methylation of the progesterone receptor PGR promoter was associated with lower levels of ER-α expression.

Other types of epigenetic regulation may be associated with the development of HR- breast cancers. For example, ER-negative tumors display hypomethylation and over-expression of several breast cancer-related genes [108],[109]. Christensen, *et al.* [110] tested 162 primary breast tumors and found that triple-negative hormone status was significantly associated with altered DNA methylation patterns in a set of 130 cancer-related genes. Although they also found trends towards increased methylation with increasing total dietary folate intake using an unsupervised clustering method, none of the 8 profiles was significantly associated with HR status. This may be due in part to a moderate skewing of the sample towards ER-positive tumors in their sample (88%) compared to the full Kaiser Permanente Northern California cancer registry (78%).

Within the field of human social genomics, there is increasing interest in gene expression profile regulation *via* neuroendocrine stress responses. Cole [111] notes that early research on the expression of stress-related genes has been difficult to replicate for several reasons, including the high level of statistical noise that comes from both measurement error and true biological variability across time, individuals, and tissues. He argues that the prior conception of “stress genes” is faulty in that “it is unlikely that any gene is regulated solely and consistently by glucocorticoids or catecholamines, and thus constitutes a pure, reliable indicator of stress uncontaminated by other regulatory influences.” Instead, he suggests taking an abstractionist approach to functional genomic data that focuses on the biological causes and consequences of gene expression, either in terms of the differential expression patterns of functionally-defined groups of genes (*i.e.*, receptor activity genes), or in terms of the common regulatory pathways that lead to differential gene expression (*i.e.*, decreased glucocorticoid receptor, GR-mediated transcription). While this approach has yet to be applied directly to the study of aggressive breast cancer subtypes and/or the associated population-level disparities, this set of molecular mechanisms are worth exploring within a weathering model.

## 4. Conclusion

In the present article, we review findings from stress biology, breast cancer epidemiology, and health disparities to understand how social and behavioral factors related to structurally rooted biopsychosocial stressors may underlie Black-White disparities in HR- breast cancer. Our review indicates a clear need to ➀ re-examine the relationships among race, social stressors, and breast cancer using more sophisticated study design, measures of stressors, and assessment of biologically distinct breast cancer subtypes; and ➁ examine the social context of race and stress, as this context could have important biological implications and yield opportunities for novel interventions to reduce breast cancer subtype disparities.

Guided by the weathering hypothesis, our model provides an important conceptual framework for generating theoretically- and empirically-driven breast cancer subtype disparities research. This research may identify important multilevel pathways for social structural conditions to differentially affect the health of disadvantaged minority populations.

The implications of such pathways could go well beyond breast cancer, as the relationships described in our conceptual model add to our general understanding of the complex ways in which social environmental conditions and the stressors that they produce may contribute to health inequalities across racial groups. They also suggest new hypotheses and methodological approaches for studying the observed racial disparity in breast-cancer subtype in the US. Research that spans disciplines is essential for developing effective interventions to prevent breast cancer disparities. Yet, positive change also depends on policy makers, social advocates, and public health practitioners supporting the conduct of this research.
